# Mitochondria-derived peptides in aging and healthspan

**DOI:** 10.1172/JCI158449

**Published:** 2022-05-02

**Authors:** Brendan Miller, Su-Jeong Kim, Hiroshi Kumagai, Kelvin Yen, Pinchas Cohen

**Affiliations:** Leonard Davis School of Gerontology, University of Southern California, Los Angeles, California, USA.

## Abstract

The mechanisms that explain mitochondrial dysfunction in aging and healthspan continue to be studied, but one element has been unexplored: microproteins. Small open reading frames in circular mitochondria DNA can encode multiple microproteins, called mitochondria-derived peptides (MDPs). Currently, eight MDPs have been published: humanin, MOTS-c, and SHLPs 1–6. This Review describes recent advances in microprotein discovery with a focus on MDPs. It discusses what is currently known about MDPs in aging and how this new understanding could add to the way we understand age-related diseases including type 2 diabetes, cancer, and neurodegenerative diseases at the genomic, proteomic, and drug-development levels.

## Mitochondria and aging

Mitochondria have been closely examined in aging because of their roles in cellular energy production, calcium homeostasis, apoptosis, and cell signaling (1). For example, mitochondrial dysfunction is linked to metabolic and oxidative damage pathology in Alzheimer’s disease and Parkinson’s disease (2–5). This is not entirely surprising because the brain is one of the most mitochondria-rich tissues and is particularly sensitive to changes in mitochondrial function. Similarly, mitochondrial dysfunction is also linked to diabetes and obesity. Recent research suggests that this dysfunction may even be causative (6–8). Likewise, mitochondrial dysfunction and mitochondrial DNA instability are highly associated with cancer (9–11). In many cancers, a phenomenon called the Warburg effect, in which these cancerous cells switch to glycolysis over oxidative respiration, has been observed (12).

Several aspects of mitochondrial dysfunction — such as mitochondrial DNA (mtDNA) mutations, nuclear DNA mutations that encode mitochondrial proteins, mitochondrial copy number, and morphological dynamics (i.e., fusion and fission) — have been studied in aging. First, mitochondria do not possess comparable DNA repair mechanisms to those found in the nucleus. Their inefficient repair mechanism combined with the proximity of the mtDNA to the electron transport chain — which generates a large amount of reactive oxygen species — promotes a high number of mtDNA mutations over a lifetime. Excessive mtDNA mutations cause an aging phenotype, as demonstrated in POLG mutator mice, which have early sarcopenia, hair graying and loss, abnormal body composition, reduced fertility, and reduced lifespan (13–15). Additional studies in worms, flies, and mice suggested that manipulating mitochondria and mitochondrial genes could increase lifespan across species (16–19). For example, increased lifespan by anywhere from 15% to 30% depending on the genetic background is observed following reduction of the gene encoding the mitochondrial enzyme CLK-1, which is required for proper mitochondrial function (*clk-1* in worms, or *Mclk1* in mice) (17, 20, 21). Yet exactly how this increase in lifespan is dependent on mitochondrial mechanisms remains unclear (22, 23).

Furthermore, mitochondrial copy number, the relative ratio of mtDNA compared with nuclear DNA, has been connected to aging. In humans, low mitochondrial copy number in peripheral blood cells is associated with poorer cognitive functioning and higher all-cause mortality (24). However, these results should be interpreted with caution. It is unclear whether changes in mitochondrial copy number in, for example, brain regions and brain cell types absolutely affect cognition. To robustly estimate the effects of mitochondrial copy number on cognitive function requires great statistical power in the form of sufficient sample size, cell dissociation, and deep phenotypic characterization. However, a recent report did implement whole-genome sequencing on 1361 human brain samples and highlighted that Alzheimer’s disease patients showed low mitochondrial copy number (25). Determining whether neuronal or glial mitochondrial copy number is driving these effects, and improving mtDNA-specific next-generation sequence analysis, are excellent objectives worth addressing in future research. The latest research has suggested the usefulness of machine learning–based approaches to improve quantification of mtDNA copy number and low-frequency variants to estimate heteroplasmy (26).

Moreover, the roles of mitochondrial morphological dynamics in aging continue to be explored, but many questions remain unanswered. Still, inhibition of fission in *Saccharomyces cerevisiae* resulted in accelerated death, an effect that was also seen in *Caenorhabditis* *elegans* and *Drosophila* *melanogaster* (27–29). Additionally, mice under calorie restriction not only lived longer but also showed increased mitochondrial length in muscle fibers (30). Likewise, in human postmortem brain samples with Alzheimer’s disease, the mitochondrial fusion proteins OPA1, MFN1, and MFN2 were significantly reduced, and levels of the mitochondrial fission protein FIS1 were significantly elevated (31).

Taken together, these findings suggest that mitochondria are central to many age-related diseases and perhaps to the fundamental aging process.

## Microproteins

Nearly 20 years ago, the Human Genome Project estimated that 20,000 to 25,000 genes encode functional proteins (32). Today, over 18,000 of these proteins have been validated by the Human Proteome Project (33). However, one element has been missed: microproteins. The term “microprotein” refers to biologically active peptides shorter than 100 amino acids (34). Bioinformatics analysis of all possible open reading frames in the human genome suggests that there may be millions of theoretical microproteins, and tens of thousands of potential microprotein mRNAs have been proposed based on ribosome profiling experiments, although most have not been detected by mass spectrometry because they are small, low-abundance, or hydrophobic (35). Indeed, thousands of microproteins have been inferred by ribosome profiling. In some ribosome profiling experiments, approximately 10,000 microproteins were identified (35). In other ribosome profiling setups with relaxed parameters, nearly half a million small open reading frames (sORFs) were identified (36). Data from several of these experiments have been added to genome-wide information on protein synthesis visualization (GWIPS-viz; https://gwips.ucc.ie/) (37), allowing researchers to explore ribosome profiling data across species, models, and experimental conditions. Nevertheless, ribosome profiling has several limitations. It is computationally challenging to detect the exact sORF undergoing translation, because codon periodicity often overlaps several sORFs. As a result, calling algorithms make two choices: throw away the reads as low-confidence, leading to false negatives; or infer active sORF translation, leading to false positives (38). Because of these limitations, hundreds of thousands of exclusive sORFs might be called across experiments, leading to reproducibility problems. To overcome these limitations, antibodies have been made against select microproteins such as the mitochondria-modifying peptides BRAWNIN, humanin, and MOTS-c (39, 40).

Detectable microproteins challenge traditional gene annotation. Most human genes have been described as monocistronic, but nearly three-quarters of microprotein sORFs detected by ribosome profiling are encoded within 5′-untranslated regions (5′-UTRs) (35, 41). As a result, many transcriptomes might not actually be monocistronic and might in fact encode multiple unique proteins ranging from dozens of amino acids (sORF) to hundreds of amino acids (large downstream coding region). One example of this phenomenon is a microprotein encoded by a sORF in the 5′-UTR of the gene encoding mitochondrial elongation factor 1 (MIEF1) (42). The MIEF1 microprotein (MIEF-1MP) and the MIEF annotated large protein act together. MIEF1-MP localizes to mitochondria — as does the larger MIEF protein — and modifies mitochondrial translation rates. Other examples include the microproteins ASDURF, BiP ORF, HJV uORF (upstream ORF), MP31, PRL-1 and PRL-2 uORF, and SEHBP, all of which have diverse functionality related to protein chaperones, ion homeostasis, and metabolic regulation (43–48). Without advances in proteomic and genomic technologies, the transcripts on which these sORFs reside would still be considered monocistronic.

Many other transcript types in both prokaryotic and eukaryotic genomes contain sORFs. Ironically, several “long noncoding RNAs” encode biologically active microproteins. The microprotein ASAP, encoded by *LINC00467*, induces age-associated colorectal cancer proliferation, while the microprotein CIP2A-BP, encoded by *LINC00665*, inhibits triple-negative breast cell invasion (49, 50). Additionally, in a comparative genomics study on almost 2000 metagenomes, approximately 4500 candidate microproteins were categorized into cell-cell communication, antimicrobial, antiphage, and adaptation activities (51). Since the gut microbiome has been connected to age-related disease progression — including Alzheimer’s disease and metabolic dysfunction (52) — the repercussions of both eukaryotic and prokaryotic microproteins are relevant to human biology.

## Mitochondrial microproteins (mitochondria-derived peptides)

**Figure 1:**
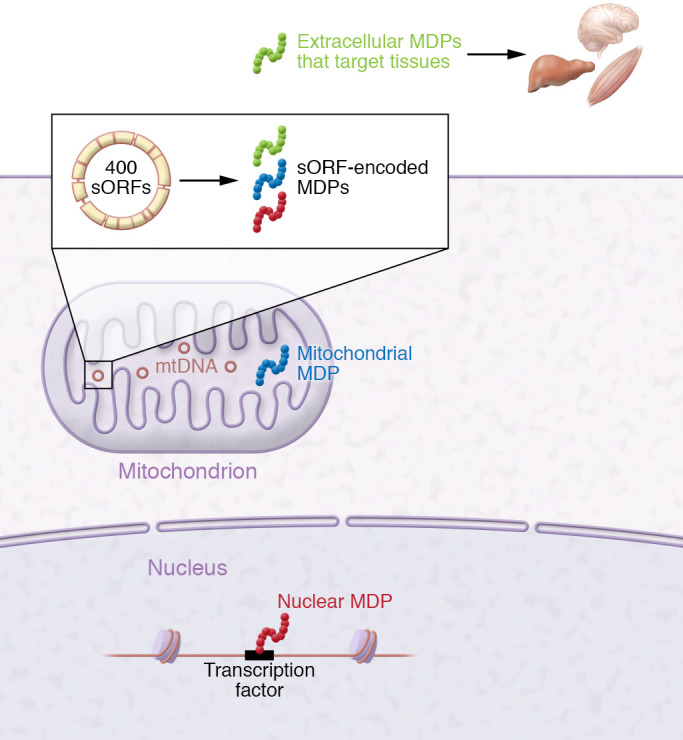
Overview of mitochondria-derived peptides. Mitochondria contain DNA with small open reading frames (sORFs) that encode functional microproteins, called mitochondria-derived peptides (MDPs). These MDPs can stay inside mitochondria, enter the cytosol, translocate to the nucleus, or be secreted extracellularly to target tissues.

Human mtDNA contains hundreds of sORFs that encode putative microproteins called mitochondria-derived peptides (MDPs). Some of these act intracellularly, while others are found in the systemic circulation and target various tissues (ref. 53 and Figure 1). The first MDP to be discovered was humanin. Humanin is a 24–amino acid peptide encoded from the 16S rRNA region of mtDNA (54). Hashimoto et al. initially cloned humanin from the resilient occipital lobe of an Alzheimer’s disease patient’s brain and found that the peptide protected against amyloid-β toxicity in neuronal cells (55). Around the same time, two additional laboratories discovered humanin as a cytoprotective peptide that binds the proapoptotic molecules IGFBP3 and BAX (56, 57). Since then, humanin has been described as a cytoprotective factor in cardiovascular, metabolic, and neurological contexts (Table 1). These effects have been in part mediated by the interaction of humanin with the tripartite receptor complex comprising gp130, WSX1, and CNTF receptor as well as with a second interacting receptor, formyl peptide receptor 2 (58). Downstream effects of this humanin cascade include activation of the AKT/ERK1/2 and STAT3 pathways (59).

After the discovery of humanin, seven additional mitochondrial microproteins were identified. Six of these, named small humanin-like peptides 1 to 6 (SHLPs 1–6), are encoded from the 16S rRNA region and share some biological features with humanin (60). For example, SHLP2 protects cells from amyloid β–induced toxicity and age-related macular degeneration (61). SHLP2 also has been characterized as a chaperone, because it bound IAPP species and blocked amyloid seeding (62). This chaperone-like activity might link its cytoprotective roles, suggesting that SHLP2 has potential as a metabolic therapeutic. Moreover, administration of SHLP2 and SHLP3 promotes mitochondrial biogenesis, reduces reactive oxygen species, and decreases mtDNA oxidation (60). Unlike these cytoprotective SHLPs, SHLP6 was shown to induce apoptosis in multiple cell lines (60). Much remains to be learned about the mechanisms of these SHLPs through future experimentation.

Another MDP that has been studied deeply over the past several years is MOTS-c, a 16–amino acid peptide encoded by a mitochondrial sORF within the 12S rRNA (63). MOTS-c was first described as an exercise mimetic peptide because it prevented weight gain in mice with high-fat diet–induced obesity, improved insulin sensitivity, and increased exercise capacity in both obese and old mice (64, 65). In addition, MOTS-c acts as a retrograde signaling molecule by translocating from mitochondria to the nucleus and binding to metabolism-regulating transcription factors (e.g., NRF1) (66). Separate reports showed that MOTS-c increased glucose uptake and stimulated glycolysis (67). These glycolysis-stimulated effects of MOTS-c were notably muted when AMPK and SIRT1 were knocked down, suggesting that AMPK and SIRT1 might be part of the action of MOTS-c (63) and might be involved in longevity.

Currently, mtDNA is annotated with 13 large mRNAs, 22 tRNAs, and 2 rRNAs. Yet since the emergence of MDPs, long noncoding RNAs, and small RNAs, mitochondrial genomic regulation appears much more complex. In their landmark paper, Mercer et al. found dozens of previously uncharacterized cleavage sites and small RNAs derived from tRNAs with unknown function (68). In another report, nearly 400 putative MDPs between 9 and 40 amino acids in silico were annotated and considered putative (69). To characterize these putative orphan MDPs, existing technology needs to be enhanced, especially ribosome profiling technology. Specifically, a mitochondrial ribosome inhibitor that stalls ribosomes at the start codon could yield additional MDPs. Overall, technical advancement in mitochondrial ribosome profiling and small peptide enrichment mass spectrometry has potential for discovery of new MDPs.

## Human mitochondrial genomics

Given that there are approximately 100 to 1000 copies of mtDNA per cell and 37 trillion cells in the human body, one human might contain nearly 2 × 10^15^ copies of mtDNA (70, 71). However, existing genomic tools are primarily designed to study nuclear DNA, as mtDNA does not undergo recombination or follow Hardy-Weinberg equilibrium. As a result, bioinformatics pipelines and genetic editing techniques for mtDNA are limited. For instance, during genome-wide association studies (GWAS), mtDNA variants are usually filtered from analytic plans. Just a few years ago, a commonly used GWAS tool called PLINK was updated to accurately estimate the effect of mtDNA variants during mtDNA-exclusive analysis (72). Yet unlike traditional GWAS, there is no gold standard method for mitochondrial GWAS (MiWAS). In GWAS, genetic population structures (genetic ancestry) are controlled by data reduction techniques such as principal component analysis. Perhaps the best illustration of this is the 2008 report by Novembre et al. in which principal component analysis on half a million DNA variants in Europeans mirrored the geography of Europe (73). In previous MiWAS reports, though, many analytic methods did not consider controls for mtDNA-specific genetic ancestry. Some of these analytic methods instead considered mitochondrial haplogroups based on mitochondrial SNPs (mtSNPs), but these haplogroup assignments are largely based on genome arrays that might lack depth (74–76). Further complicating MiWAS is that population cohorts often have extremely variable mtSNP frequencies.

Nevertheless, recent reports highlighted significant effects of frequent mtSNPs on human phenotypes in large population cohorts. For example, a report by Yonova-Doing et al. included a phenome-wide mtDNA-phenotype association analysis on 260 candidates in over 300,000 individuals (77). They found significant associations between mtDNA variants and type 2 diabetes (T2D), multiple sclerosis, height, and liver and renal function. Likewise, Kraja et al. found several mtDNA variants that associated with multiple metabolic traits in 45 combined cohorts (78). Independent reports on smaller cohorts noted associations between mtDNA variation and neurodegeneration including Parkinson’s disease, Alzheimer’s disease, and eye disease (79–81). While MiWAS can reveal meaningful mitochondrial genomic regions, its statistical limitations necessitate experimental validation.

Validating MiWAS associations experimentally is incredibly challenging because of the fundamental problem that mtDNA editing lacks fidelity. Whereas laboratories can edit single nuclear nucleotides with CRISPR, mtDNA cannot be edited with similar precision (82). Thus, MiWAS is rarely followed up with comprehensive functional experimentation, although in vitro models called cybrids, whereby mtDNA is depleted from a cell line and then replaced by donor mtDNA, have been used (83, 84). Cybrid approaches have revealed functional effects of certain mtDNA variants, but they are limited by the fact that other mtDNA variants are transferred to the parent cell line. To bypass problems with cybrids and mtDNA gene editing, overexpression or recombinant administration of mitochondrially encoded proteins has been considered. In cells that harbored mtATP6 mutations, overexpression of mtATP6 restored homeostasis (85). Similarly, a SNP in MOTS-c leading to a MOTS-c variant called K14Q raises the risk of T2D in Japanese men, and unlike WT MOTS-c, K14Q failed to protect from metabolic dysfunction in vivo, proving it to be a bioinactive form of the hormone (86). Moreover, a separate mtSNP within the humanin sORF associated with lower circulating humanin peptide and with more severe cognitive decline, suggesting that the variant affects translation of the humanin transcript (87), leading to decreased neuroprotection. In the coming years, precise mtDNA editing, whole-genome sequencing of large population cohorts, and functional mitochondrial gene annotation can all help validate MiWAS associations.

## MDPs in age-related diseases

**Figure 2:**
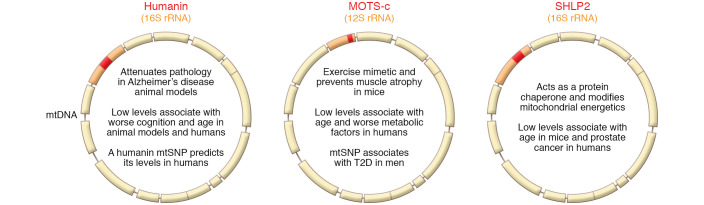
Mitochondria-derived peptides in age-related disease. Three MDPs have been studied in the context of age-related diseases: humanin, MOTS-c, and SHLP2. Humanin has been shown to mitigate Alzheimer’s disease pathology in rodents, and its levels and genetic variation associate with age and cognition. MOTS-c has been described as an exercise mimetic and prevents muscle atrophy in mice, and its levels and genetic variation associate with age and type 2 diabetes (T2D). SHLP2 functions as a mitochondrial modulator and protein chaperone, and its levels associate with age and prostate cancer.

MDPs have been extensively studied in the context of aging. Age-related diseases such as T2D, coronary endothelial dysfunction, and Alzheimer’s disease have been associated with lower MOTS-c or humanin levels in plasma (refs. 63, 88, 89, and Figure 2). In humans, circulating MOTS-c and humanin were downregulated in patients with T2D (90), and circulating MOTS-c levels were negatively correlated with BMI, fasting insulin levels, and homeostatic model assessment of insulin resistance (91). T2D was also associated with the MOTS-c variant K14Q (mentioned above), a naturally occurring m.1382A>C polymorphism (rs111033358) that changes the 14th amino acid of MOTS-c from lysine to glutamine (i.e., K14Q). In human skeletal muscle, MOTS-c expression correlated with slow-twitch muscle fiber gene expression (89); the C allele carriers of the m.1382A>C polymorphism were associated with more fast-twitched fibers (92); and circulating MOTS-c levels predicted myostatin levels in men (93). These human associations were corroborated in vivo when skeletal muscle atrophy was attenuated by MOTS-c during aging in mice fed a high-fat diet (65, 93).

Several studies suggest that MOTS-c and humanin are possible biomarkers for cardiovascular disease. People with endothelial dysfunction, a strong risk factor for cardiovascular events (94), displayed low MOTS-c and humanin levels. Likewise, circulating MOTS-c and humanin levels positively correlated with coronary endothelial function (95, 96). A follow-up study by Ikonomidis et al. demonstrated that T2D patients with low circulating MOTS-c levels (<167 ng/mL) exhibited a more than 3-fold higher risk of cardiac events than those with high MOTS-c levels (97). Similarly, Cai et al. demonstrated that circulating humanin levels at baseline were an independent risk factor for major adverse cardiac events in patients with angina (98). In vivo and in vitro experiments support these observations. Wei et al. reported that MOTS-c prevented vascular calcification by activating the AMPK signaling pathway and suppressing angiotensin II type I receptor (AT1) and endothelin B expression in rats treated with vitamin D3 and nicotine (99). Moreover, humanin has increased expression of KLF2, an essential transcriptional regulator of endothelial function, and regulated endothelial nitric oxide synthase and endothelin-1 in vitro. Comparably, humanin has suppressed endothelial dysfunction and atherosclerosis progression in vivo (100). These findings suggest that MOTS-c and humanin are associated with cardiovascular disorders via endothelin and vasoactive regulation.

Since humanin was originally detected in the occipital lobe of a patient with Alzheimer’s disease, it has been tested as a therapeutic agent in several models of neurodegeneration. For example, humanin has prevented synaptic loss in hippocampal neurons and reduced astrocytic inflammation (101). In double- and triple-transgenic mouse models of Alzheimer’s disease, an analog of humanin called S14G-humanin (or S14G-HNG) improved cognition (102, 103). In humans, patients with Alzheimer’s disease had lower humanin levels in cerebrospinal fluid than controls (104), and humanin genetic variation was linked to cognition, as the naturally occurring m.2706A>G polymorphism (rs2854128) within the humanin sORF associated with accelerated cognitive aging in African Americans (87). Altogether, these observations suggest that humanin could be a potential biomarker and therapeutic target for cognitive decline and neurological disorders such as Alzheimer’s disease.

Studies have demonstrated associations between MDPs and cancer. Xiao et al. showed that prostate cancer (PCa) patients had low circulating SHLP2 levels (105). They suggested that circulating SHLP2 levels may be useful for predicting the risk of PCa in patients undergoing biopsy (105). Separately, several studies suggest that humanin ameliorates negative side effects of chemotherapy (106–108). In addition, Lue et al. demonstrated that S14G-HNG treatment in mouse models not only decreased negative side effects of chemotherapy but also decreased metastasis of cancer cells (109).

## MDPs in longevity

In addition to their ability to attenuate age-related diseases, MDPs have promoted lifespan and healthspan. In fact, circulating humanin levels decreased with age in both mice and human plasma (88). Intriguingly, further studies found that this decrease also occurred in monkeys but not in the long-lived naked mole rat, which is a model of negligible senescence and healthy aging (104). Human offspring of centenarians, who have a greater chance of living to be 100 years old, even displayed elevated levels of circulating humanin compared with age-matched controls without family history of exceptional longevity (104). These associations have been studied intensively using two separate experimental paradigms. The first was to utilize the power of *C*. *elegans* to generate transgenic worms overexpressing humanin. Humanin is the best-conserved MDP and is found in as diverse species as humans, naked mole rats, and nematodes (65, 110). Overexpression of humanin sufficiently increased lifespan, and this was dependent on FOXO (104). These data supported our previous work that found that humanin is regulated by and also regulates the insulin/IGF pathway, the upstream signaling pathway of FOXO (111). Additionally, humanin has increased autophagy in cells, and this increase in autophagy was also required for the lifespan extension in the transgenic worms (112–114). The second approach was to initiate a longevity experiment in mice in which we injected middle-aged (18-month-old) female mice with humanin twice a week (104, 115). Although lifespan was not increased — likely because of humanin’s short half-life of approximately 20 minutes — healthspan measures such as memory and metabolic parameters improved (87, 104, 115). Thus, humanin is sufficient to increase lifespan and healthspan in model organisms, and an optimized dosing of humanin may lead to increases in lifespan in more complex organisms.

Exercise has been shown to have many benefits preventing and attenuating age-related diseases such as sarcopenia and cognitive decline. MOTS-c — previously described as an exercise mimetic — may also show similar benefits (116). In humans, circulating MOTS-c levels decrease with age, as does humanin. But unlike humanin, MOTS-c has been shown to increase its levels in skeletal muscle (89). Moreover, MOTS-c was found to have effects on lifespan itself. Reynolds et al. found that intraperitoneal administration of 15 mg/kg MOTS-c three times a week starting at 23.5 months of age caused a trend toward increased lifespan that did not quite reach significance (65). As with the lifespan study in humanin-treated mice, this lack of significant increase may have been due to a suboptimal dosing for a lifespan study and relatively short half-life of circulating MOTS-c. On the other hand, these mice did have significant improvement in grip strength, gait, and physical performance, demonstrating an increase in healthspan in MOTS-c–treated animals.

Although other MDPs have been discovered, they have not been examined in the context of general aging, except for SHLP2. Circulating SHLP2 levels did decrease with age in both female and male mice, but no lifespan experiments have been conducted (60). Given SHLP2’s ability to protect against models of macular degeneration, its correlation with PCa risk, and its effect on senescent cells, it is easy to imagine that SHLP2 could also affect lifespan and healthspan similarly to other MDPs (61, 105, 117). A complete list of the physiological significance of SHLP and other MDPs is shown in Table 1.

## The future of MDP science

As technology improves, more MDPs and nuclear-encoded microproteins will be discovered and functionalized. Technologies such as ribosome profiling and small peptide–enriched peptidomics represent enormous opportunities for microprotein discovery pipelines. Currently, there are no chemicals that stall mitochondrial ribosomes at their start codons, in contrast to harringtonine and lactimidomycin, which stall cytoplasmic ribosomes at their start codons (35). Despite the need to identify these mitochondria-specific start codon inhibitors, there has still been high interest in mitochondrial ribosome profiling and specific protocols described that might guide further development (118). The capacity to stall mitochondrial ribosomes at their start codons would reveal MDPs that are preferentially translated by mitochondrial ribosomes. Moreover, enhancing the purity of existing ribosome profiling methods might also reveal mitochondrial transcripts that undergo translation by cytoplasmic ribosomes.

Ribosome-centric technologies are nevertheless snapshots of translation. Peptide evidence of translation and stability in the form of mass spectrometry is crucial. While invaluable innovations in small peptide mass spectrometry have pushed the microprotein field forward over the last decade, there is still room for optimization. Many microproteins possess intrinsically disordered regions or hydrophobicity that make their detection difficult for existing mass spectrometry methods. New methods to capture these difficult-to-detect MDPs and nuclear-encoded microproteins would greatly inform the field.

Moreover, innovative discovery methods might differentiate humanin-like sequences encoded by nuclear mtDNA segments (NUMTs). For instance, Eltermaa et al. reported that NUMT genetic variation within proximity of MTRNR2L2 (a humanin NUMT) and MTRNR2L13 (a separate humanin NUMT) nominally associated with coronary function, albeit these associations did not survive statistical correction (44). Whether these NUMTs functionally encode humanin-like sequences remains unknown, but if indeed they do, then their levels have implications for measuring humanin levels considering their sequence identity to mtDNA-encoded humanin.

The ability to sensitively detect microproteins will have enormous clinical implications. Microproteins might serve as novel biomarkers and diagnostics for diseases that lack such predictive measures. Targeted assays using immunological techniques (e.g., ELISA) are logical, but they lack the high throughput capacity for the omics boom. Measuring MDPs across biological tissues in large-scale tissue banks would also address the degree to which disease, mitochondrial copy number, and mitochondrial morphological dynamics affect MDP levels. Moreover, microprotein detection at the large-scale omics levels would promote interdisciplinary collaboration and data sharing, possibly leading to rapid clinical translation. Some microproteins might even serve as therapeutic targets. In fact, the MOTS-c analog CB4211 has been posed as a therapeutic target in clinical trials for nonalcoholic steatohepatitis and obesity (NCT03998514; ClinicalTrials.gov). Information about this MOTS-c analog is publicly available. Its intellectual property was licensed by CohBar Inc. and used to develop potent analogs of MOTS-c, for which the US Patent and Trademark Office has granted a patent (US 11,111,271). As the field discovered the function of many miRNAs, it was suggested that a new therapeutic era was unfolding (119). Indeed, miRNAs have informed the field about biological processes that could very well lead to viable interventions. In the same vein, both nuclear-encoded and mitochondrially encoded microproteins might represent a new era of therapeutics, genomics, and proteomics.

## Figures and Tables

**Table 1:**
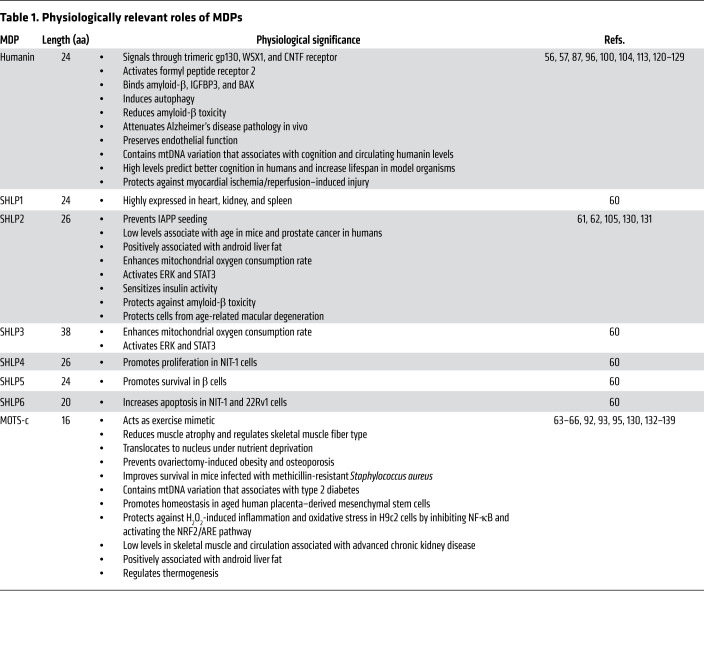
Physiologically relevant roles of MDPs

## Version 1 05/02/2022

Electronic publication
